# Efficacy of fibrin sealant for bedside pleurodesis in patients with prolonged air leak after lung cancer surgery: a comparative study

**DOI:** 10.3389/fsurg.2025.1722846

**Published:** 2026-01-09

**Authors:** Kaifang Pan, Xin Gu, Zhe Chen, Jiahao Yu, Kai Xie, Jiangjiang Liu, Jingkang Qi, Bin Wang, Haifeng Xia

**Affiliations:** 1 Department of Thoracic Cardiovascular Surgery, The Fourth Affiliated Hospital of Soochow University, Suzhou, China; 2 Department of General Practice, The Fourth Affiliated Hospital of Soochow University, Suzhou, China

**Keywords:** fibrin sealant, glucose solution, non-small cell lung cancer, pleurodesis, prolonged air leak

## Abstract

**Background:**

Prolonged air leak (PAL) represents a significant clinical problem after lung resection surgery, frequently causing extended hospital stays. Although several treatments exist, their effectiveness often remains limited. Fibrin sealant has attracted attention as a potential alternative due to its biocompatible properties, but strong evidence from controlled studies supporting its use for bedside pleurodesis remains insufficient. This study was conducted to compare the effectiveness and safety of fibrin sealant vs. 50% glucose solution in managing PAL following pulmonary resection for non-small cell lung cancer (NSCLC).

**Methods:**

We performed a retrospective analysis of NSCLC patients who developed PAL (lasting ≥5 days) after surgery between January 2021 and May 2025. Patients received either fibrin sealant or 50% glucose solution for bedside pleurodesis. To address potential selection bias, we employed propensity score matching (1:1 ratio) based on key clinical characteristics. Primary outcomes included success rate after initial intervention and time to chest tube removal. Secondary outcomes focused on complication rates.

**Results:**

After matching, 74 pairs were available for analysis. The fibrin sealant group showed significantly better outcomes, with a higher success rate after the first intervention (56.8% vs. 24.3%; OR = 4.08, 95% CI: 2.02–8.24, *p* < 0.001) and shorter time from intervention to median chest tube duration (6.0 days vs. 9.5 days, *p* < 0.001). All patients in the fibrin sealant group achieved resolution within three interventions, while some in the glucose group required up to seven procedures. Complication rates were similar between groups (16 cases each, *p* > 0.05), with no infection-related complications observed in the fibrin sealant group.

**Conclusion:**

For patients with PAL after NSCLC resection, bedside pleurodesis using fibrin sealant appears more effective than 50% glucose solution. It offers better initial success rates, significantly reduces air leak duration, and demonstrates a comparable safety profile. These findings support considering fibrin sealant as a primary non-surgical treatment option for this challenging complication.

## 1 Introduction

Postoperative prolonged air leak (PAL), defined as air leakage continuing for more than five days after thoracic surgery, remains a difficult clinical problem. This condition typically results from bronchopleural or alveolar-pleural fistulas and associates strongly with longer hospital stays and increased postoperative complications. Reported PAL rates after pulmonary surgery range from 5.4% to 9.5% (1, 2). Furthermore, recent studies have continued to identify specific risk factors for PAL following anatomic resections such as segmentectomy (3, 4). Treatment approaches include observation, suction drainage, surgical re-intervention, endobronchial valve placement, and chemical pleurodesis (5–8). Despite these options, managing PAL effectively continues to challenge clinicians, with many treatments providing unsatisfactory results.

Among pleurodesis agents, 50% glucose solution represents a relatively safe and economical option that may help reduce hospital stay duration (9). In comparison, the use of fibrin sealant for postoperative pleurodesis has been less documented, with supporting evidence mainly coming from case reports (10, 11). Fibrin sealant works by mimicking the final stage of the coagulation cascade to form a stable fibrin clot, providing a mechanical advantage through direct sealing of pleural defects. Given the limited comparative evidence, we designed this study to systematically evaluate and compare the efficacy and safety of fibrin sealant against 50% glucose solution for bedside pleurodesis in patients with PAL following NSCLC resection.

## 2 Materials and methods

### 2.1 Study design and patient selection

This retrospective study reviewed patients who underwent pulmonary resection for NSCLC at the Fourth Affiliated Hospital of Soochow University between January 2021 and May 2025. The Institutional Review Board approved the study protocol (Ref: 20251247), and waived individual informed consent due to the retrospective nature. We included patients who developed air leakage lasting ≥5 days postoperatively and received bedside pleurodesis. Patients were categorized into two groups based on the pleurodesis agent used: fibrin sealant or 50% glucose solution. The selection of the pleurodesis agent was determined through a standardized discussion between the attending surgeon and the patient. This discussion comprehensively covered the characteristics of both options: for fibrin sealant, its off-label use, potential for higher efficacy, and significant out-of-pocket cost; and for 50% glucose solution, its established safety profile and minimal cost. The final choice balanced clinical judgment with the patient's informed preference, a potential source of bias that was mitigated by propensity score matching.

### 2.2 Data collection and outcome measures

We collected demographic and baseline characteristics (age, gender, body mass index, smoking history), operative details (surgical approach, procedure type), and comorbidities (emphysema, hypertension) from medical records. Primary outcomes were: (1) success rate after initial pleurodesis attempt, and (2) time from intervention to chest tube removal. Secondary outcomes included incidence of postoperative complications graded as Clavien-Dindo class II or higher.

### 2.3 Bedside pleurodesis protocol

PAL was confirmed if air leakage persisted beyond the fifth postoperative day. Pleurodesis was initiated after radiographic confirmation of lung expansion with less than 20% atelectasis.

50% Glucose Group: Patients received 5 mL of 2% lidocaine followed by 60 mL of 50% glucose solution through the chest tube. The tube was then clamped and elevated, with careful patient repositioning to distribute the agent.

Fibrin sealant Group: A commercial 10 mL porcine fibrin sealant kit was used. The fibrinogen and thrombin components were injected sequentially into the pleural space, followed by saline flushes and patient repositioning.

Chest tube management protocols were identical for both groups. Removal criteria included absence of air leak during coughing and 24-h drainage volume below 100 mL, confirmed by radiography. Patients with persistent leaks underwent re-intervention at approximately five-day intervals.

### 2.4 Statistical analysis

To address potential selection bias, we performed propensity score matching (1:1) using a caliper width of 0.1. The propensity score was estimated via a logistic regression model that included the following pre-specified covariates: age, gender, body mass index (BMI), smoking history, presence of emphysema, hypertension, and type of resection (wedge resection vs. segmentectomy/lobectomy). The balance of covariates between groups was assessed using absolute standardized mean differences (SMDs), with an SMD <0.1 indicating adequate balance. The success of the matching procedure in achieving balance for all covariates is detailed in Supplementary Table 1. Continuous variables were summarized as mean ± standard deviation or median with interquartile range, compared using Student's *t*-test or Mann–Whitney *U* test as appropriate. Categorical variables were expressed as counts and percentages, compared using Chi-square or Fisher's exact test. A two-tailed *p*-value < 0.05 indicated statistical significance. Analyses used SPSS (version 27.0) and R (version 4.4.1). A *post-hoc* power analysis was performed using GPower software (version 3.1) for the comparison of complication rates, a key secondary outcome with a non-significant result, employing Fisher's exact test with a two-sided alpha of 0.05. To control for potential residual confounding after propensity score matching, multivariable regression analyses were performed for the primary outcomes. A multivariable logistic regression model was constructed for the outcome of success after the first intervention, adjusting for age, gender, BMI, smoking status, emphysema, hypertension, and type of surgery. And a multivariable Cox proportional hazards model was constructed for the outcome of time to chest tube removal. The results are presented as adjusted odds ratios (OR) or hazard ratios (HR) with their 95% confidence intervals (CIs).

## 3 Results

### 3.1 Patient characteristics

Table 1 summarizes the baseline demographic and clinical characteristics of the patients before and after propensity score matching. Initial screening identified 190 eligible patients with PAL: 84 treated with fibrin sealant and 106 receiving 50% glucose. Propensity score matching created 74 well-matched pairs. The balance of all covariates was substantially improved after matching, as detailed in Supplementary Table 1. In the overall cohort of 190 patients who developed PAL, the distribution of surgical procedures was as follows: 46 patients (24.2%) had undergone wedge resection, 144 patients (75.8%) had undergone anatomic resection (segmentectomy or lobectomy). Among the anatomic resections, segmentectomy was performed in 98 patients (51.6% of the total cohort), and lobectomy was performed in 46 patients (24.2% of the total cohort). This indicates that anatomic resections, particularly segmentectomy, constituted the majority of cases complicated by PAL in our study population. The absolute SMDs for the majority of covariates (7 out of 9) were below the 0.1 threshold. Although the SMDs for 'smoking status' (SMD = 0.11) and “wedge resection” (SMD = 0.13) were slightly above 0.1, they represented a marked reduction from the pre-match imbalances (SMD = 0.47 and 0.18, respectively) and were deemed acceptable for comparative analysis. Comparative analysis showed no significant differences in baseline characteristics (*p* > 0.05 for all), indicating successful control of potential confounders.

**Table 1:** Demographic data of patients.

Characteristics	Before matching			After matching		
	FS (*n* = 84)	50%GS (*n* = 106)	*p*-value	FS (*n* = 74)	50%GS (*n* = 74)	*p*-value
Age (yr)	59.62 ± 14.48	61.55 ± 16.51	0.400	59.81 ± 15.43	60.54 ± 16.65	0.78
Sex			0.042			0.67
Male	72 (85.7)	78 (73.6)		62 (83.8)	60 (81.1)	
Female	12 (14.3)	28 (26.4)		12 (16.2)	14 (18.9)	
Body mass index	22.02 ± 2.71	22.83 ± 5.01	0.18	22.09 ± 2.87	22.27 ± 2.98	0.71
Smoking status	48 (57.1)	36 (34.0)	0.01	38 (51.4)	34 (45.9)	0.51
Laterality			0.461			1.00
Left	32 (38.1)	46 (43.4)		32 (43.2)	32 (43.2)	
Right	52 (61.9)	60 (56.6)		42 (56.8)	42 (56.8)	
Surgery			0.210			0.64
Wedge resection	24 (28.6)	22 (20.8)		20 (27.0)	16 (21.6)	
Segmentectomy &Lobectomy	60 (71.4)	84 (79.3)		54 (73.0)	58 (78.4)	
Comorbidity	44 (52.4)	62 (58.5)	0.400	42 (56.8)	44 (59.5)	0.74
High blood pressure	24 (28.6)	38 (35.8)	0.288	24 (32.4)	22 (29.7)	0.72
Emphysema	24 (28.6)	34 (32.1)	0.602	24(32.4)	22(29.7)	0.72

FS, fibrin sealant; 50%GS, 50% glucose solution.

### 3.2 Efficacy outcomes

**Figure 1:**
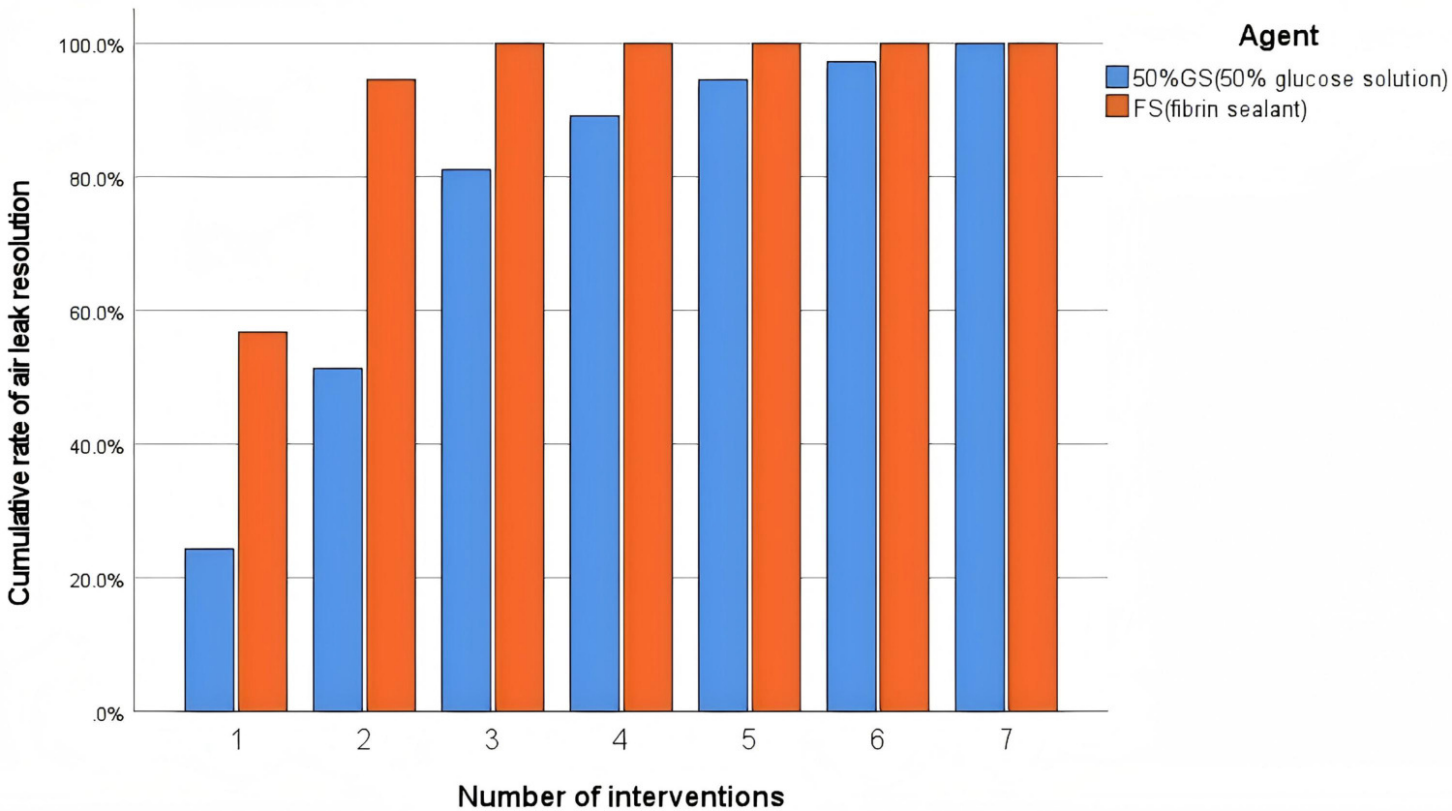
Comparison of efficacy between 50%GS and FS.

**Figure 2:**
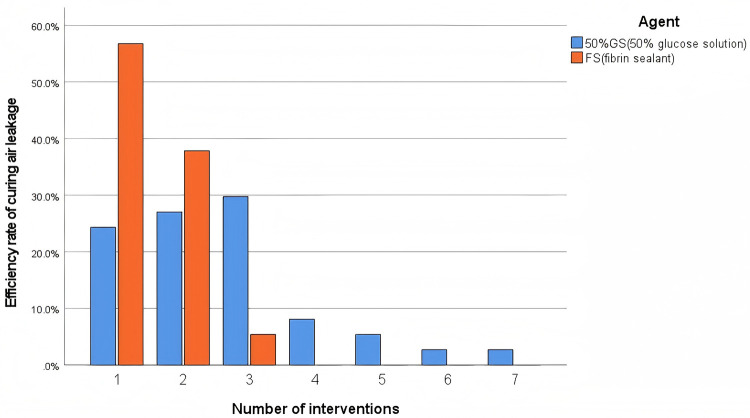
Number of interventions required for air leak resolution.

**Figure 3:**
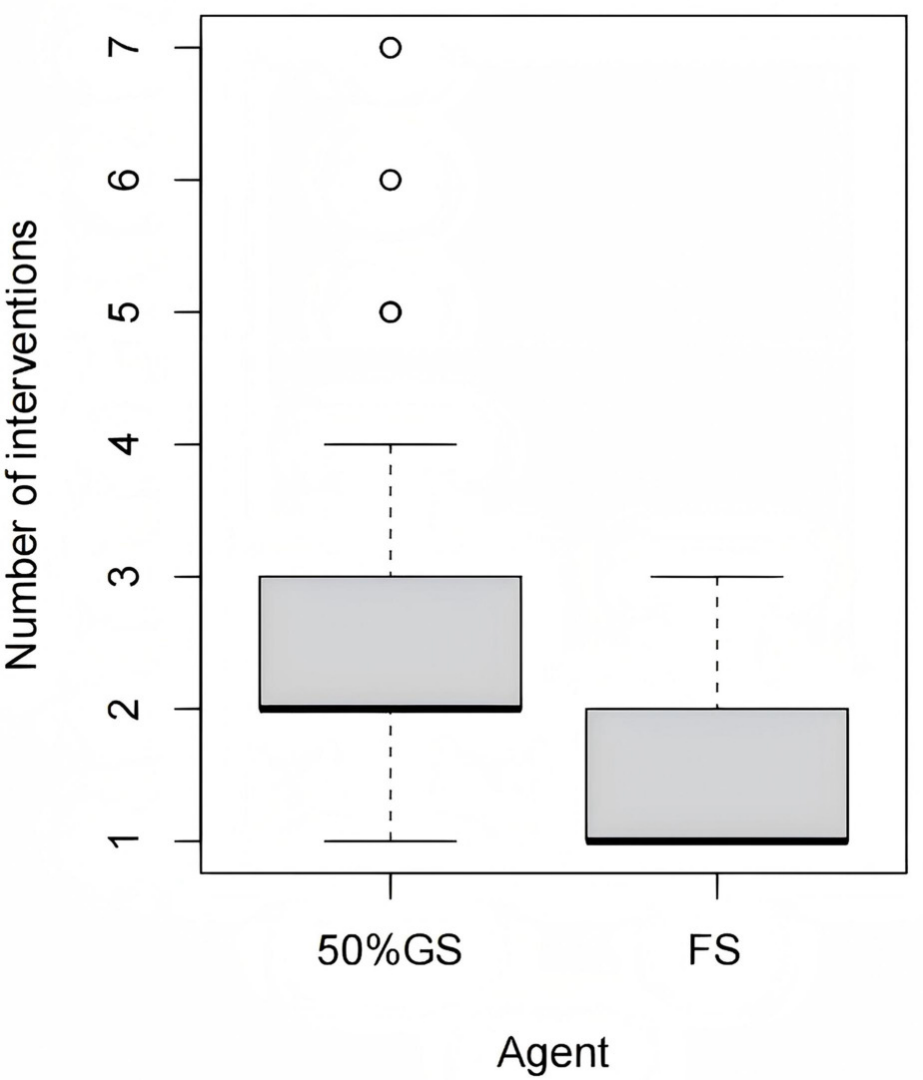
Comparison of the number of interventions required between FS and 50%GS.

The primary efficacy outcomes are presented in Table 2. The fibrin sealant group demonstrated significantly superior efficacy. The success rate after the first intervention was more than twice as high in the fibrin sealant group compared to the 50%GS group [56.8% [42/74] vs. 24.3% [18/74]; OR = 4.08, 95% CI: 2.02–8.24, *p* < 0.001]. Consequently, the fibrin sealant group required significantly fewer interventions [median: 1 [IQR: 1, 2] vs. 2 [IQR: 1.75, 3.00], *p* < 0.001]. This difference in the number of interventions required for air leak resolution is also visually depicted in Figures 1, 2. Figure 3 shows that all patients in the fibrin sealant group achieved resolution within three interventions, while some in the 50%GS group required up to seven procedures.

**Table 2:** Outcomes after bedside pleural fixation in the FS group and 50%GS group.

Outcomes	FS (*n* = 74)	50%GS (*n* = 74)	*p*-value
Number of interventions	1.00 (1.00, 2.00)	2.00 (1.75, 3.00)	<0.001
Days of diversion	6.00 (3.00, 8.00)	9.50 (4.00, 13.25)	<0.001
Complications	16 (21.6)	16 (21.6)	1.00
Pleural effusion	11 (14.9)	8 (10.8)	
Pulmonary atelectasis	5 (6.8)	5 (6.8)	
Incisional infection	0	2 (2.7)	
Pus thorax	0	1(1.4)	

FS, fibrin sealant; 50%GS, 50% glucose solution.

**Figure 4:**
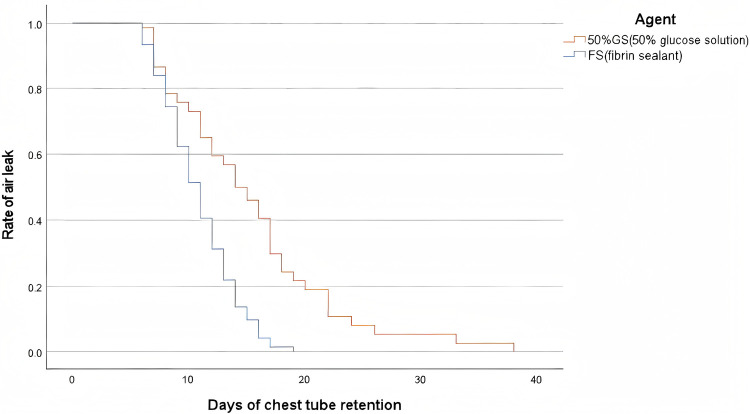
Kaplan-Meier curve for prolonged air leak risk.

Furthermore, the time from the first intervention to chest tube removal was significantly shorter in the fibrin sealant group [median: 6.0 days [IQR: 3.0, 8.0] vs. 9.5 days [IQR: 4.0, 13.25], *p* < 0.001], as shown in Table 2. This finding is visually supported by the Kaplan–Meier curve in Figure 4, which illustrates the cumulative rate of chest tube removal over time, demonstrating a clear advantage for the fibrin sealant group. Among the subset of patients with a successful first intervention, the time from that intervention to complete resolution of the air leak was similar between groups (3.62 ± 1.61 days vs. 2.94 ± 1.06 days, *p* > 0.05).

### 3.3 Safety outcomes

**Figure 5:**
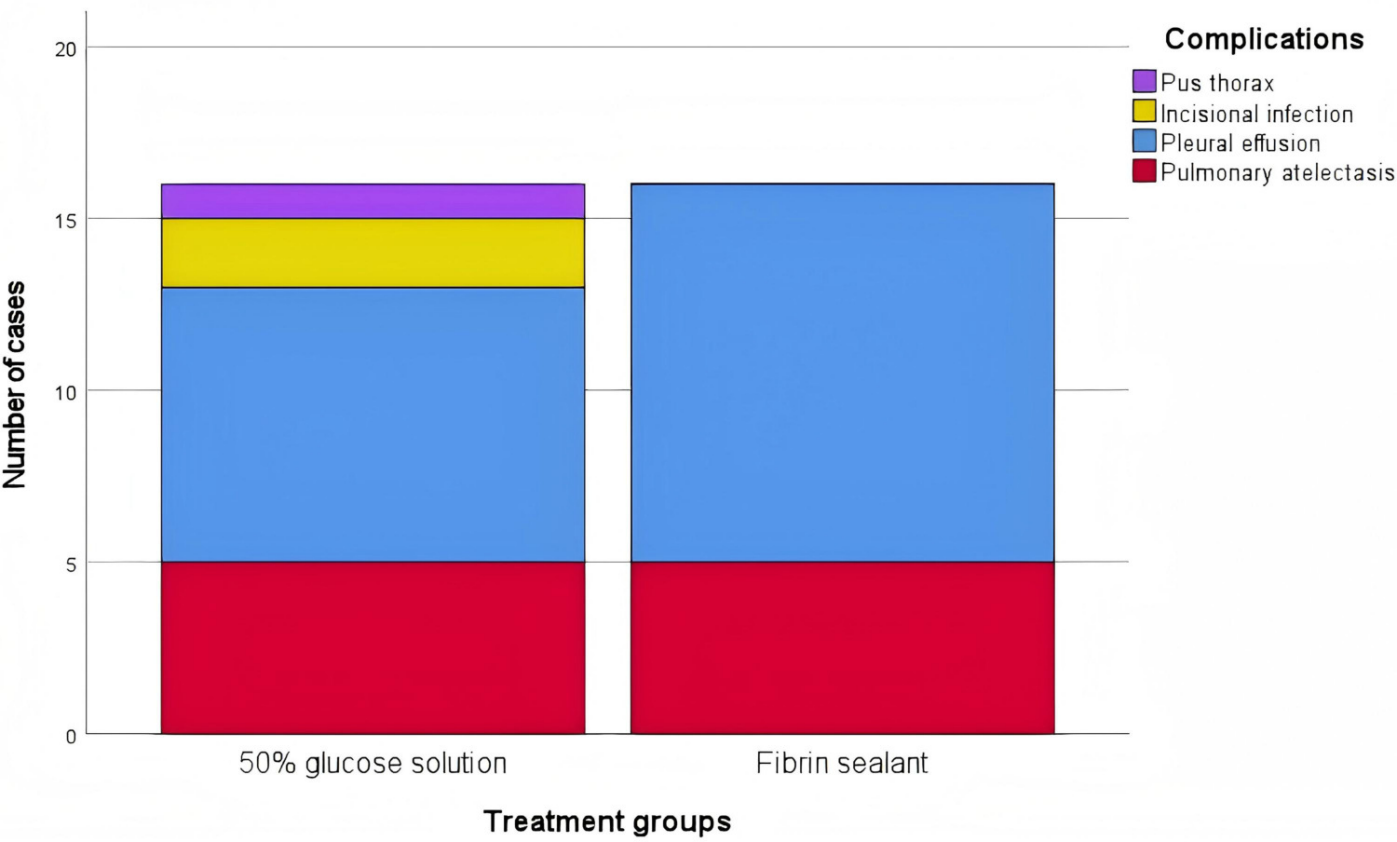
Spectrum and frequency of complications by treatment group.

Complication rates are detailed in Table 2. The overall complication rates were similar between groups (16 cases each, *p* > 0.05). The spectrum and frequency of specific complications by treatment group are further illustrated in Figure 5. A *post-hoc* power analysis indicated that the study had limited power (46%) to detect a difference smaller than 15% in complication rates; however, the identical observed rates suggest any undetected difference is likely minimal. The fibrin sealant group had 11 pleural effusions and 5 atelectasis events. The glucose group reported 8 pleural effusions, 5 atelectasis events, 2 surgical site infections, and 1 empyema case. No infectious complications or empyema occurred in the fibrin sealant group.

### 3.4 Sensitivity analyses for residual confounding and E-value analysis

**Figure 6:**
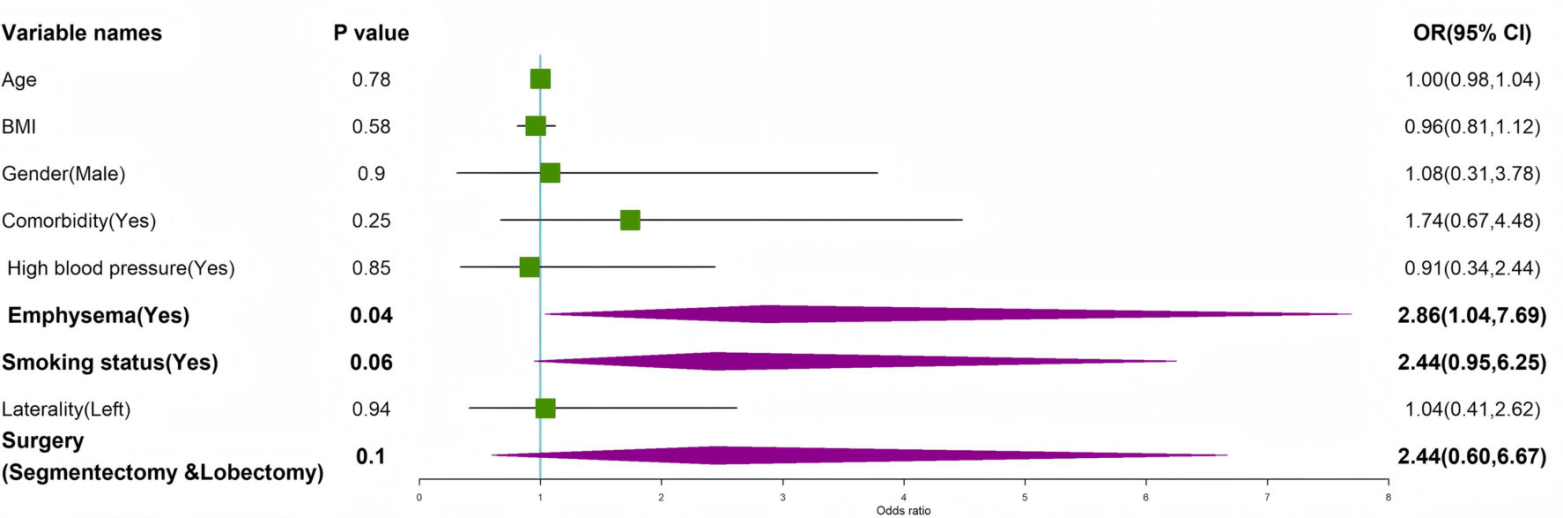
Univariable logistic regression of factors influencing first intervention efficacy in the fibrin sealant group.

**Figure 7:**
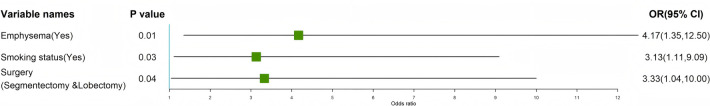
Multivariable logistic regression of factors influencing first intervention efficacy in the fibrin sealant group.

**Figure 8:**
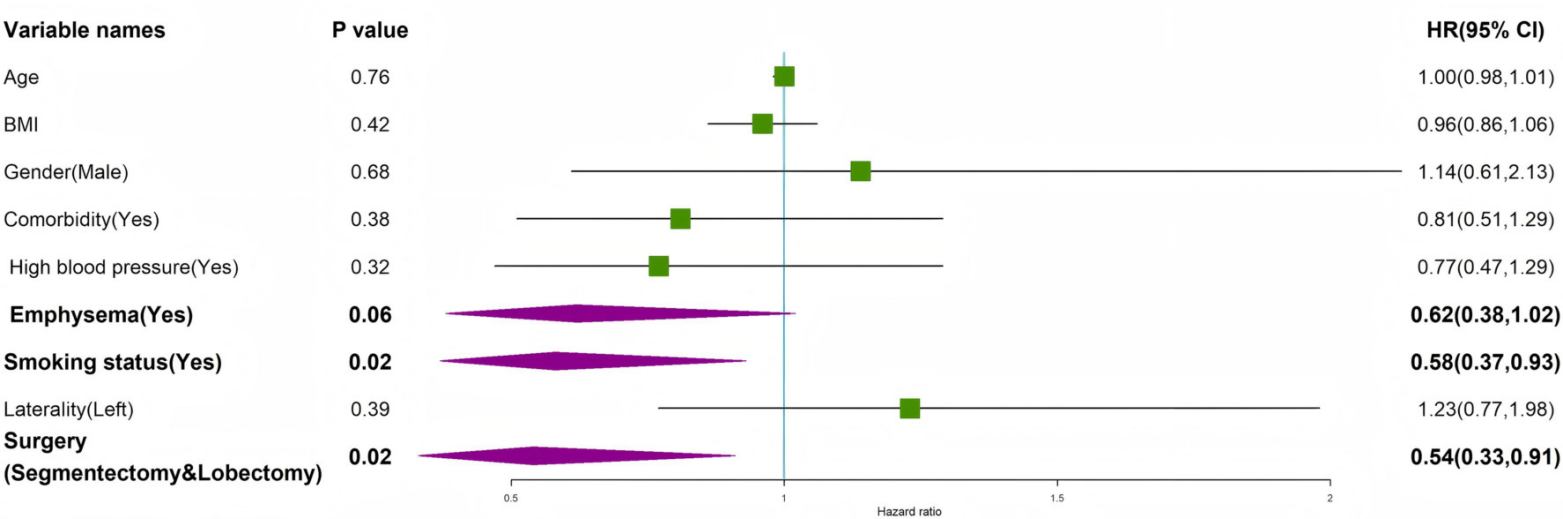
Univariable Cox regression analysis of factors influencing time to chest tubve removal.

Given minor residual imbalances in smoking status and surgery type after matching, we performed multivariable regression analyses to adjust for these and other clinically relevant factors. The results of the univariable analyses are presented in Figures 6 and 8. The results of the multivariable logistic regression for success after the first intervention are presented in Table 3 and illustrated in Figure 7. For the outcome of success after the first intervention, multivariable logistic regression analysis confirmed that the treatment group remained a strong and independent predictor of success (adjusted OR = 4.67, 95% CI: 2.35–9.28, *p* < 0.001). Furthermore, the presence of emphysema, active smoking status, and having undergone segmentectomy or lobectomy (vs. wedge resection) were also identified as significant independent predictors of a successful first intervention.

**Table 3:** Multivariable logistic regression analysis of factors associated with success after the first intervention.

Variable	Adjusted odds ratio	95% Confidence interval	*p*-value
Treatment group (FS vs. 50% GS)	4.67	2.35–9.28	<0.001
Emphysema (Yes)	4.17	1.35–12.50	0.01
Smoking status (Yes)	3.13	1.11–9.09	0.03
Surgery (Segmentectomy & Lobectomy)	3.33	1.04–10.00	0.04

FS, fibrin sealant; 50%GS, 50% glucose solution.

**Figure 9:**
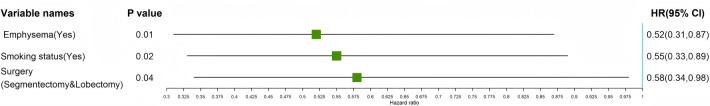
Multivariable Cox regression analysis of factors influencing time to chest tube removal.

Similarly, the results of the multivariable Cox proportional hazards model for time to chest tube removal are shown in Table 4 and Figure 9. Similarly, for time to chest tube removal, a multivariable Cox proportional hazards model yielded consistent findings for the treatment effect (adjusted HR = 2.55, 95% CI: 1.78–3.65, *p* < 0.001). In this model, emphysema, smoking, and larger resection type were associated with a longer time to tube removal. E-value analyses indicated that unmeasured confounders would need strong associations to explain away the observed effects, supporting the robustness of our conclusions.

**Table 4:** Multivariable cox regression analysis of factors associated with time to chest tube removal.

Variable	Adjusted hazard ratio	95% Confidence interval	*p*-value
Treatment group (FS vs. 50% GS)	2.55	1.78–3.65	<0.001
Emphysema (Yes)	0.52	0.31–0.87	0.01
Smoking status (Yes)	0.55	0.33–0.89	0.02
Surgery (Segmentectomy & Lobectomy)	0.58	0.34–0.98	0.04

FS, fibrin sealant; 50%GS, 50% glucose solution.

An HR >1 indicates a factor associated with earlier chest tube removal.

## 4 Discussion

Our comparative analysis indicates that bedside pleurodesis using fibrin sealant provides a more effective management strategy for PAL following NSCLC resection compared to conventional 50% glucose solution. The data show that fibrin sealant not only offers substantially higher initial success rates but also significantly reduces chest tube duration, while maintaining a comparable safety profile with possible advantages in preventing infectious complications.

The improved efficacy of fibrin sealant likely stems from its different mechanism of action. Unlike glucose, which primarily acts as an irritant to induce pleural inflammation and subsequent adhesion, fibrin sealant functions as an immediate sealant. Its ability to form a stable fibrin polymer network—similar to the body's natural coagulation process—provides a prompt physical barrier against air leakage (12, 13). This direct mechanical sealing explains the significantly better first-attempt success rate, offering a quicker solution compared to the slower inflammatory process associated with glucose pleurodesis.

Our results support previous research on fibrin sealants in thoracic surgery (14, 15). And provide stronger comparative evidence specifically for postoperative PAL management. The median time from pleurodesis to chest tube duration of 6.0 days in our fibrin sealant group, while longer than the postoperative 3-day period reported in studies focusing on intraoperative prevention (16, 17), appears clinically reasonable. This difference in the baseline starting point (postoperative day 0 vs. pleurodesis intervention day, which is ≥postoperative day 5) is critical for interpretation. This difference likely reflects our specific patient population—those with established PAL, who typically experience more complex and prolonged air leaks (1, 2, 18). Thus, our findings are particularly relevant for clinicians managing patients who have already developed this complication.

The safety profile of fibrin sealant appeared favorable. The complete absence of wound infections and empyema in the fibrin sealant group, compared to their occurrence in the glucose group, suggests a potential clinical advantage. The high-glucose environment created by intrapleural glucose installation, even in non-diabetic patients, has been previously associated with increased infection risk (9, 19–21). Additionally, the lack of significant chest pain reports in the fibrin sealant group-a known side effect of glucose pleurodesis-further supports its tolerability.

An important point requiring further discussion is whether the absence of infectious complications in the fibrin sealant group, in contrast to the glucose group, is attributable to the inherent properties of the agents or to potential confounding factors (e.g., baseline comorbidities, drain duration before intervention). Although propensity score matching balanced key baseline characteristics (e.g., age, gender, BMI, smoking history, emphysema, and surgical approach), unmeasured variables could theoretically influence outcomes. However, given that PAL was defined as air leak lasting ≥5 days and pleurodesis was initiated under standardized criteria after confirming lung expansion, the pre-intervention drainage duration is likely similar between groups. Moreover, the matched groups showed no significant differences in comorbidities that might predispose to infections. The absence of infections in the fibrin sealant group is consistent with its biocompatible and sealed nature, which may reduce bacterial entry, whereas glucose solution could promote microbial growth. Thus, while residual confounding cannot be entirely excluded, the evidence suggests that the difference in infection rates is more likely explained by the distinct biological properties of the agents.

Our data corroborate previous findings that emphysema, smoking history and the extent of pulmonary resection pose higher risks for PAL (3, 4, 22). In our cohort, approximately three-quarters of all PAL cases occurred after anatomic resections, with segmentectomy alone accounting for over half of the cases. This is likely attributable to the more extensive dissection and larger raw parenchymal surface area associated with these procedures, which can challenge tissue healing and increase the potential for air leakage.

From a practical perspective, although fibrin sealant incurs higher direct costs than glucose, its significantly better first-attempt success rate and the associated reduction in time to chest tube removal (by a median of 3.5 days) may lead to shorter hospital stays. While this study was not designed as a cost-effectiveness analysis, it is plausible that the savings from the abbreviated hospitalization and the avoidance of repeated procedures could offset the initial cost of the sealant. This potential economic advantage deserves consideration in treatment decisions and should be formally evaluated in future cost-effectiveness studies to guide broader clinical adoption.

### 4.1 Limitations

Several limitations should be acknowledged. First, the retrospective design, despite propensity score matching, limits causal inference compared to randomized trials. The agent selection based on patient preference and economic factors remains a potential source of bias. Second, the sample size, while adequate for primary outcomes, may have been underpowered to detect small differences in complication rates. Nevertheless, the identical overall complication rates observed provide preliminary evidence of comparable safety. Third, exclusion of bronchopleural fistula relied on intraoperative assessment without routine postoperative bronchoscopic confirmation, possibly including minor fistulas that might respond poorly to pleurodesis. Meanwhile, the fixed five-day re-interval, while standardizing care, might not represent optimal timing for all patients. Finally, future randomized controlled trials are warranted to validate these findings and to formally assess the cost-effectiveness of adopting fibrin sealant as a first-line therapy.

## 5 Conclusion

For patients with PAL after NSCLC resection, bedside pleurodesis using fibrin sealant proves more effective and safe than 50% glucose solution. It significantly improves initial success rates, shortens air leak duration, and shows a favorable safety profile with potential protective effects against infections. The robustness of these findings is supported by sensitivity analyses and E-value calculations, indicating resistance to both measured and unmeasured confounding. These findings support considering fibrin sealant as a first-line non-surgical treatment for this difficult complication. Future randomized controlled trials should validate these observations and thoroughly evaluate cost-effectiveness.

## Data Availability

The original contributions presented in the study are included in the article/Supplementary Material, further inquiries can be directed to the corresponding author.
