# Supplementary material for: Efficacy of fibrin sealant for bedside pleurodesis in patients with prolonged air leak after lung cancer surgery: a comparative study
Source: Front Surg. 2026 Jan 9;12:1722846. doi: 10.3389/fsurg.2025.1722846 (PMC12827594; doi:10.3389/fsurg.2025.1722846)
Supplement: Supplementary file 1 [file Table1.docx]

Supplementary Table 1. Balance of Baseline Characteristics Before and After Propensity Score Matching

| Characteristics | Before matching | | | After matching | | |
| --- | --- | --- | --- | --- | --- | --- |
|  | FS  (n=84) | 50%GS  (n=106) | SMD | FS  (n=74) | 50%GS  (n=74) | SMD |
| Age (yr) | 59.62±14.48 | 61.55±16.51 | 0.12 | 59.81±15.43 | 60.54±16.65 | 0.05 |
| Sex |  |  |  |  |  |  |
| Male | 72(85.7) | 78(73.6) | 0.30 | 62(83.8) | 60(81.1) | 0.07 |
| Female | 12(14.3) | 28(26.4) |  | 12(16.2) | 14(18.9) |  |
| Body mass index | 22.02±2.71 | 22.83±5.01 | 0.20 | 22.09±2.87 | 22.27±2.98 | 0.06 |
| Smoking status | 48(57.1) | 36(34.0) | 0.47 | 38(51.4) | 34(45.9) | 0.11 |
| Laterality |  |  |  |  |  |  |
| Left | 32(38.1) | 46(43.4) | 0.11 | 32(43.2) | 32(43.2) | 0.00 |
| Right | 52(61.9) | 60(56.6) |  | 42(56.8) | 42(56.8) |  |
| Surgery |  |  |  |  |  |  |
| Wedge resection | 24(28.6) | 22(20.8) | 0.18 | 20(27.0) | 16(21.6) | 0.13 |
| Segmentectomy &Lobectomy | 60(71.4) | 84(79.3) |  | 54(73.0) | 58(78.4) |  |
| Comorbidity | 44(52.4) | 62(58.5) | 0.12 | 42(56.8) | 44(59.5) | 0.05 |
| High blood pressure | 24(28.6) | 38(35.8) | 0.15 | 24(32.4) | 22(29.7) | 0.06 |
| Emphysema | 24(28.6) | 34(32.1) | 0.07 | 24(32.4) | 22(29.7) | 0.06 |

Abbreviations: FS, Fibrin Sealant; 50%GS, 50%Glucose Solution; SMD, Standardized Mean Difference.
